# Supplementary figures and images for: Comprehensive analysis of iron utilization by Mycobacterium tuberculosis
Source: PLoS Pathog. 2020 Feb 18;16(2):e1008337. doi: 10.1371/journal.ppat.1008337 (PMC7058343; doi:10.1371/journal.ppat.1008337)

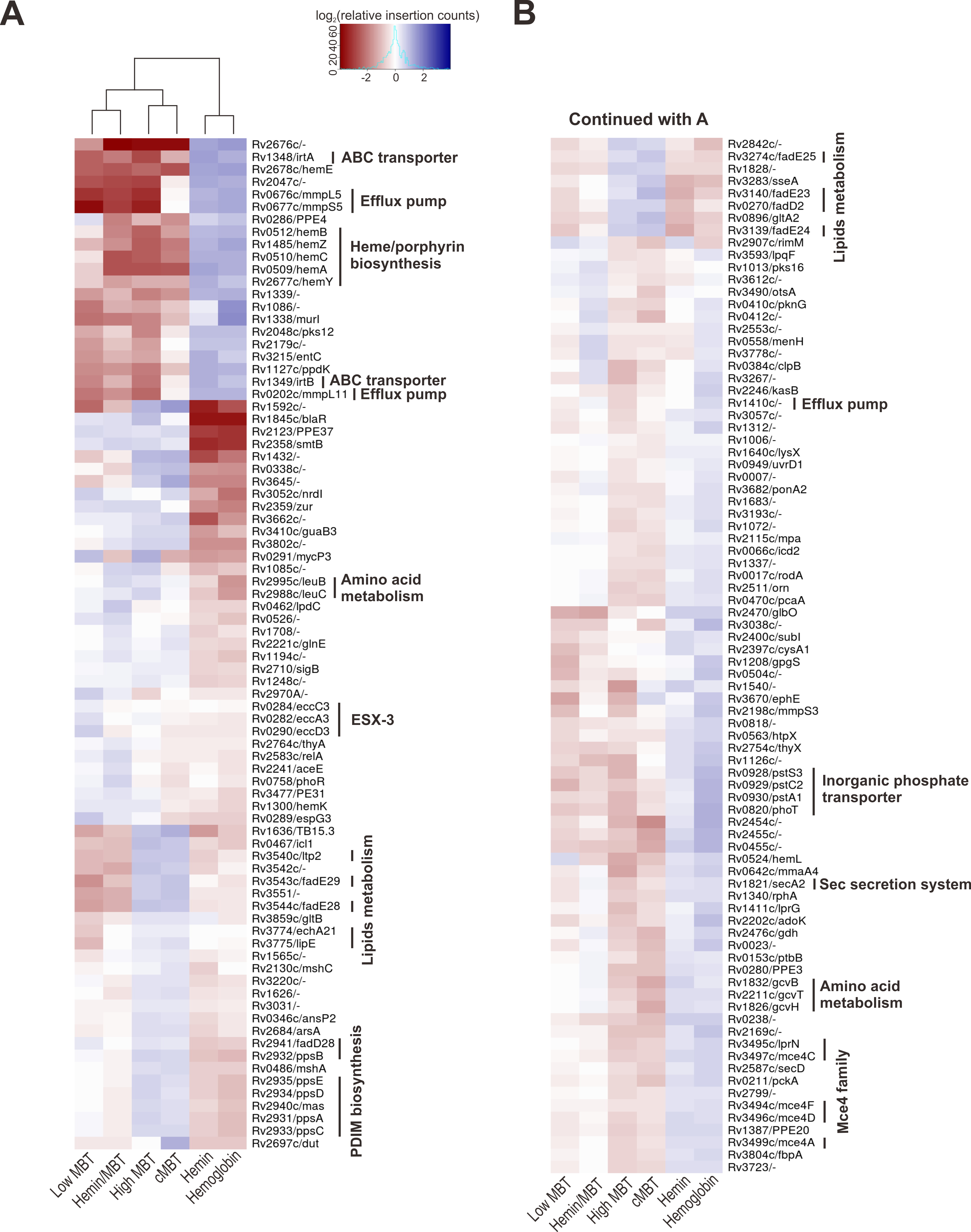

Supplement: S1 Fig — ANOVA was used to identity the 165 genes which exhibited statistically significant changes in fitness under at least one of the tested iron conditions, after correction for false discovery rates. The mean normalized insertion count across replicates is calculated for each gene, for each of the 6 conditions. Then a log-fold-change is calculated for each condition relative to the mean count across all the conditions. The color 'red' means the counts in one condition are lower than the other conditions on average, suggesting a greater requirement for that gene in that condition, and 'blue' means insertion counts are higher than average, suggesting it is less required. The dendrogram shows the hierarchical clustering of the conditions (columns) using complete-linkage clustering. Gene insertion count profiles (rows) are also clustered using the hclust package in R, and gene pathway associations are indicated. (TIF) [file ppat.1008337.s001.tif]

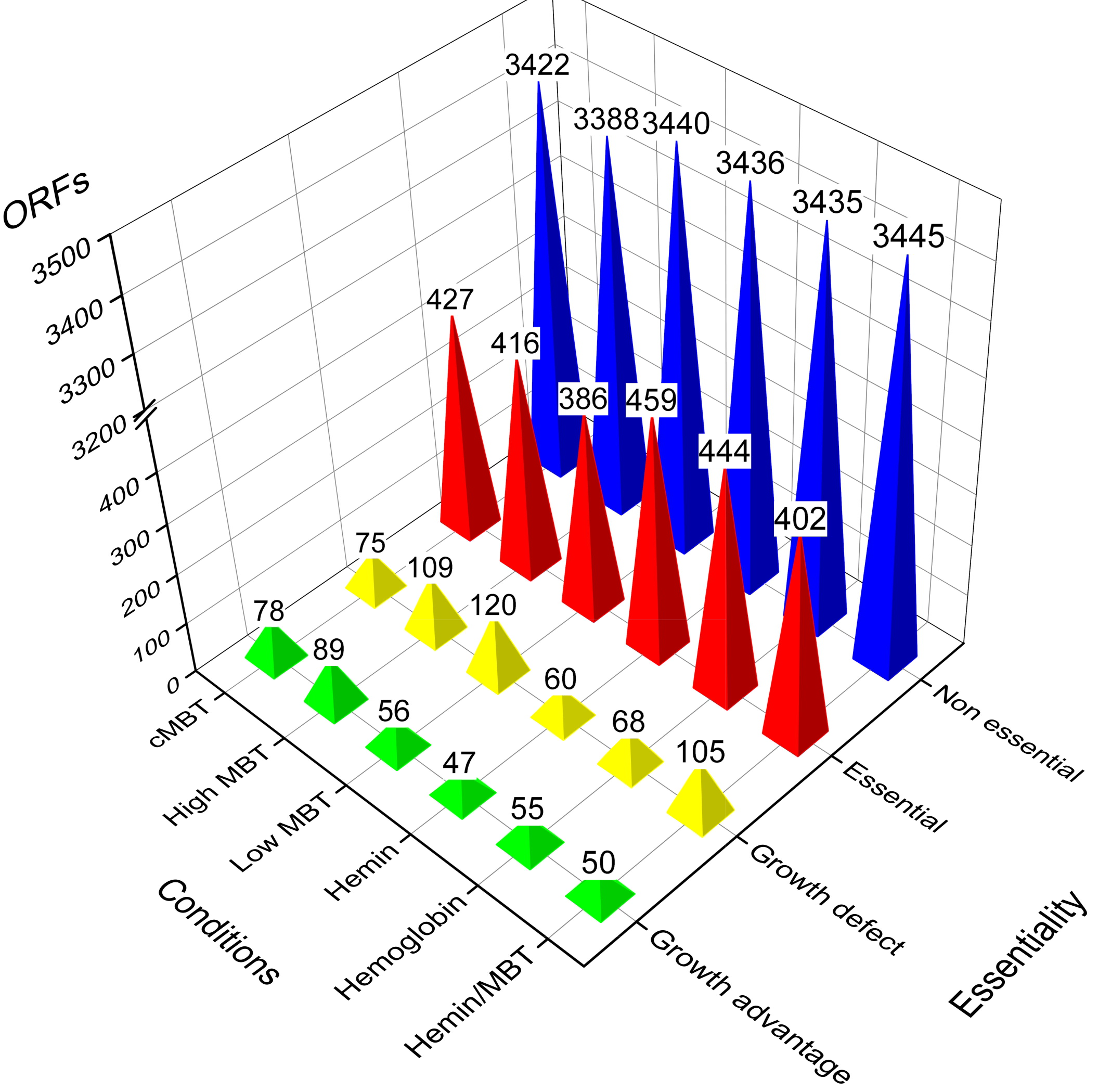

Supplement: S2 Fig — The essentiality of Mtb genes was classified using the HMM analysis. The number of genes in each class is indicated for each iron condition. (TIF) [file ppat.1008337.s002.tif]

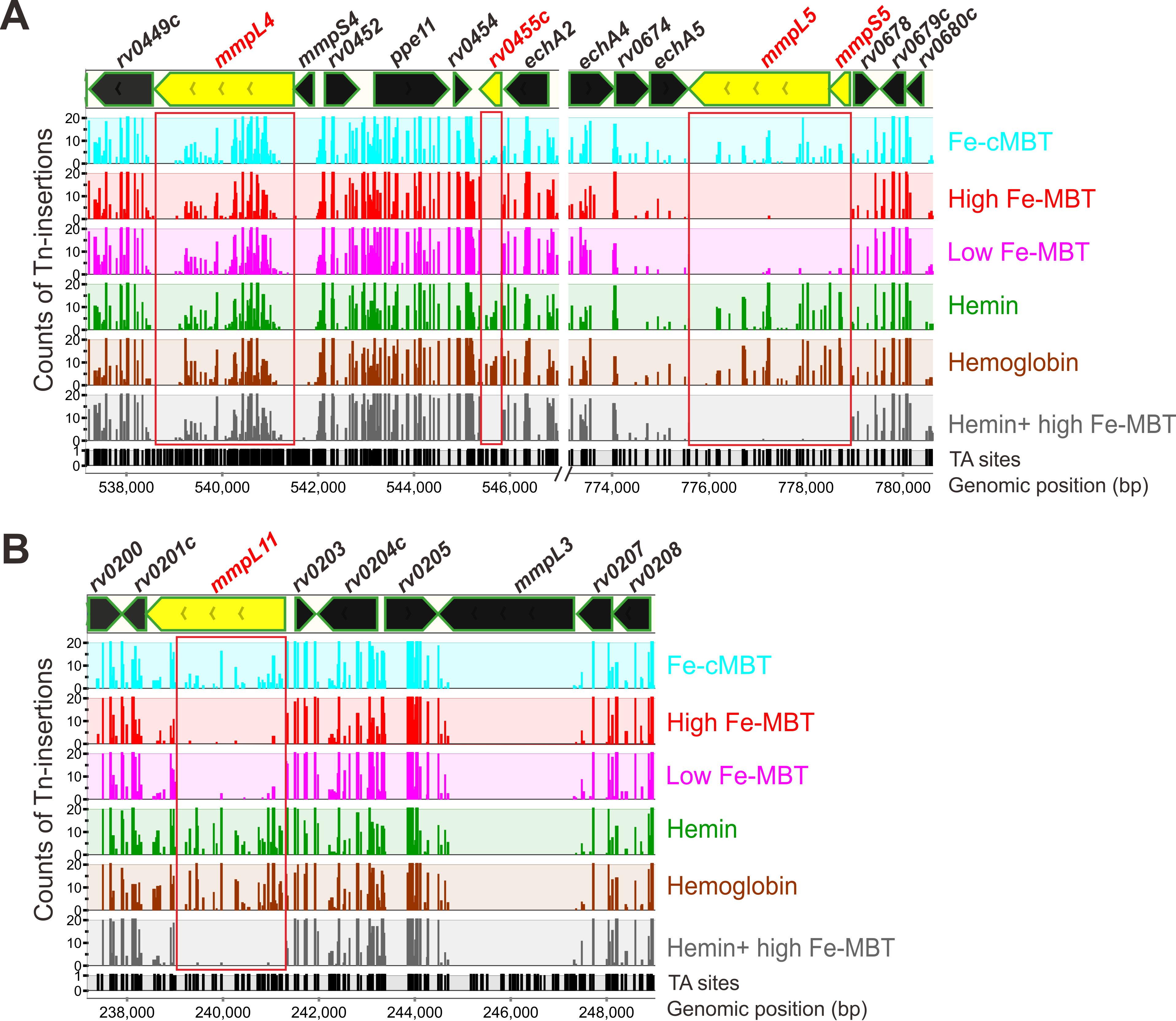

Supplement: S3 Fig — Profiles of the transposon insertions in Mtb genomic regions including mmpL4, rv0455c, mmpL5/S5 (A) and mmpL11 (B). MmpL3 is essential under all conditions consistent with previous results [82]. The iron conditions are indicated by different colors. The y-axis (0, 10, 20) represents the counts of the Tn-insertions and x-axis represents the genomic position (bp). Potential TA dinucleotide insertions sites are indicated in black. Regions containing genes of interest are boxed in red. Plots were generated using MochiView [46]. (TIF) [file ppat.1008337.s003.tif]

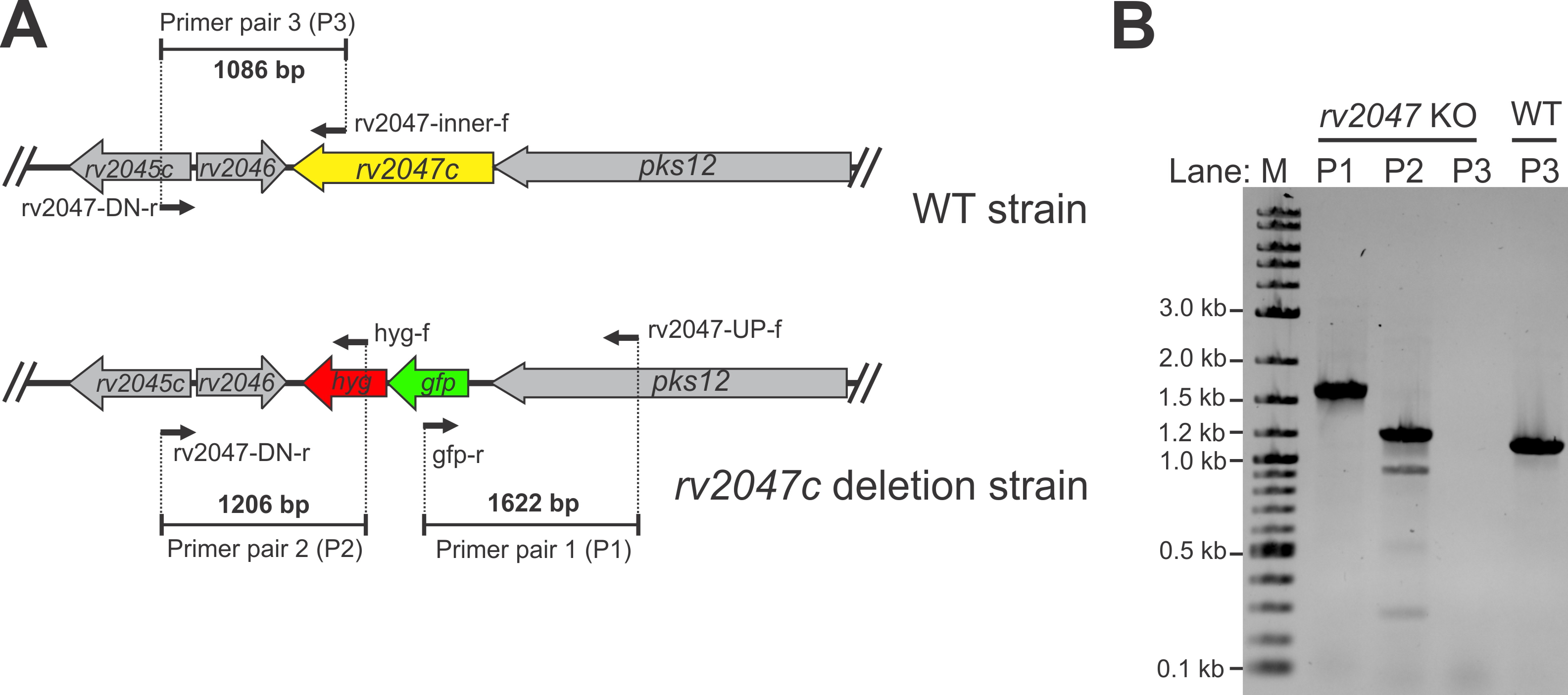

Supplement: S4 Fig — (A) Schematic representation of the Mtb H37Rv rv2047c genomic regions and PCR performed to validate deletion of rv2047c. (B) PCR using primers (S3 Table) to validate rv2047c knock out (KO) mutants in the avirulent Mtb strain mc26230 (H37Rv ΔRD1 ΔpanCD). (TIF) [file ppat.1008337.s004.tif]

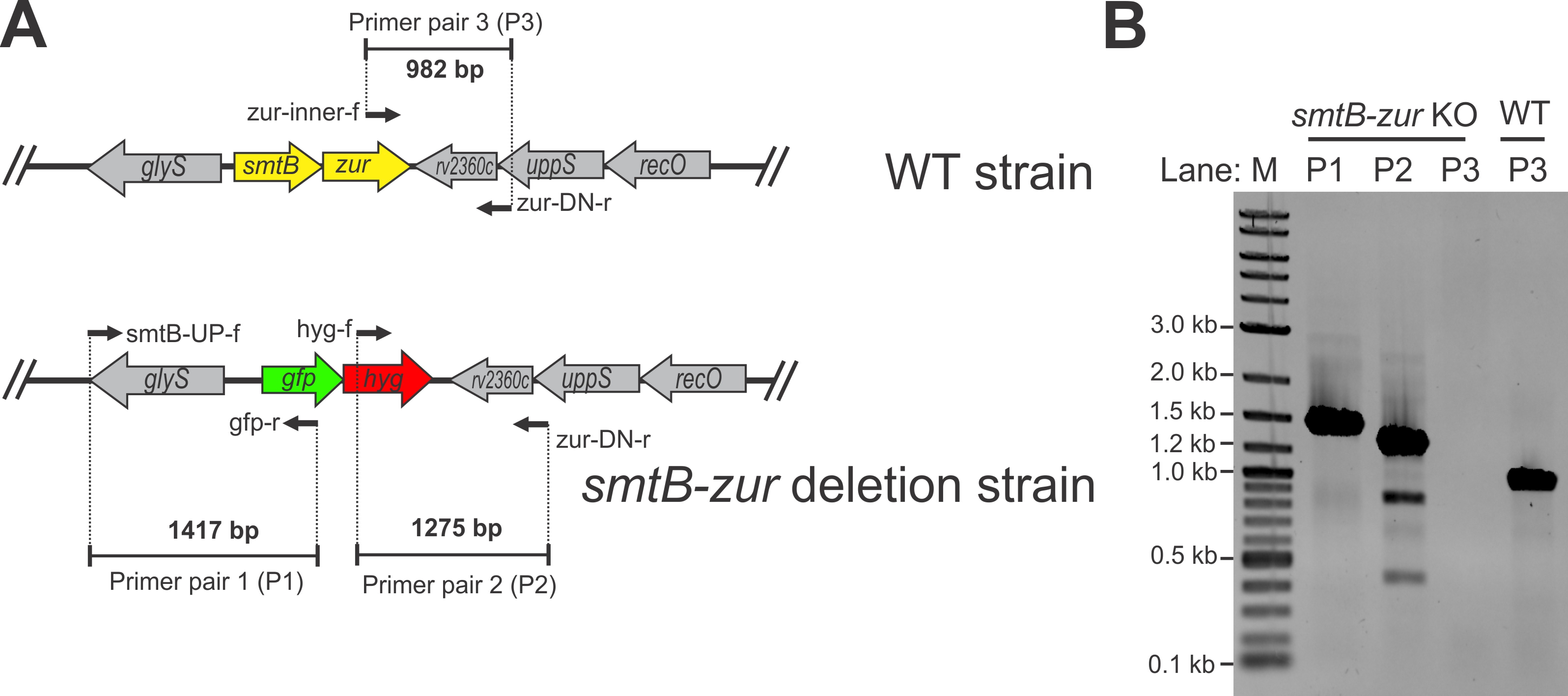

Supplement: S5 Fig — (A) Schematic representation of the Mtb H37Rv ΔsmtB-zur genomic regions and PCR performed to validate deletion of smtB-zur. (B) PCR using primers (S3 Table) to validate ΔsmtB-zur knock out (KO) mutants in the avirulent Mtb strain mc26206 (H37Rv ΔpanCD ΔleuCD). (TIF) [file ppat.1008337.s005.tif]

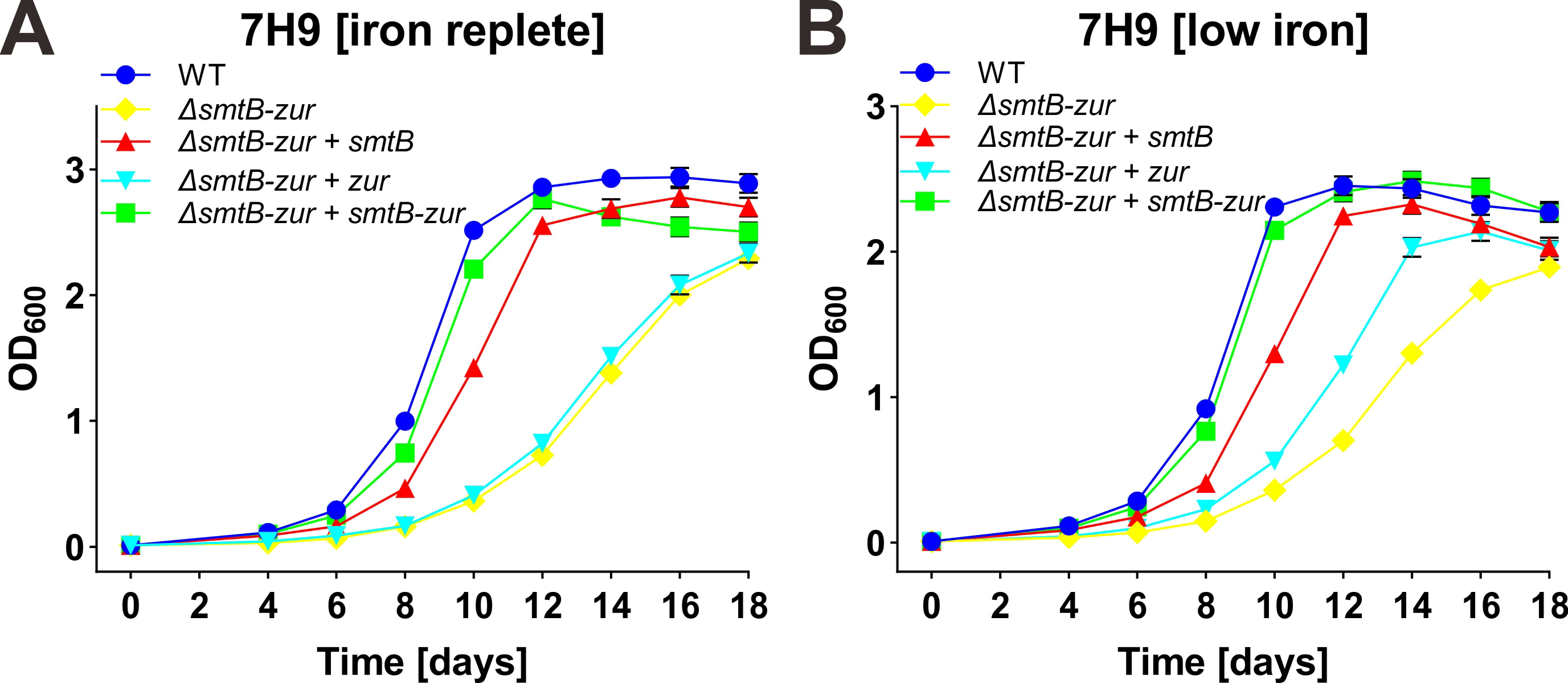

Supplement: S6 Fig — Growth assays of wild-type M. tuberculosis mc26206, ML2277 (ΔsmtB-zur deletion mutant), ML2278 (ΔsmtB-zur complemented with smtB), ML2279 (ΔsmtB-zur complemented with zur) and ML2280 (ΔsmtB-zur complemented with smtB-zur) under iron-replete (150 μM Fe3+) 7H9 medium (A) and low-iron (less than 0.1 μM Fe3+) 7H9 medium (B), respectively. The Mtb cells were grown in self-made low-iron 7H9 medium for 7 days to deplete intracellular iron before growth assays. The initial OD600 of all the cultures is 0.01. Error bars represent standard deviations from the mean of results from biological triplicates. (TIF) [file ppat.1008337.s006.tif]
