# Supplementary material for: Comprehensive analysis of iron utilization by Mycobacterium tuberculosis
Source: PLoS Pathog. 2020 Feb 18;16(2):e1008337. doi: 10.1371/journal.ppat.1008337 (PMC7058343; doi:10.1371/journal.ppat.1008337)
Supplement: S1 Table — (PDF) [file ppat.1008337.s007.pdf]

Strain	Relevant genotypes and description	Source or reference
E. coli DH5α	*recA1; endA1; gyrA96; thi; relA1; hsdR17(rK-;mK+); supE44; ϕ80ΔlacZΔM15; ΔlacZYA-argF; UE169*	[2]
M. tuberculosis $mc^2$6230	H37Rv derivative; Δ*RD1* Δ*panCD*; avirulent	[3]
M. tuberculosis $mc^2$6206	H37Rv derivative; Δ*leuCD* Δ*panCD*; avirulent	[4]
M. tuberculosis ML2256	$mc^2$6230 derivative; Δ*rv2047c*::hyg^R (*rv2047c* deletion)	This study
M. tuberculosis ML2257	$mc^2$6230 derivative; Δ*rv2047c*::hyg^R, L5 attB::pML4211 (P_{imyc}::*rv2047c*), kan^R	This study
M. tuberculosis ML2300	$mc^2$6230 derivative; Δ*mmpL4*::*loxP*	#
M. tuberculosis ML2301	$mc^2$6230 derivative; Δ*mmpL5*::*loxP*	#
M. tuberculosis ML2302	$mc^2$6230 derivative; Δ*mmpL4*::*loxP*, Δ*mmpL5*::*loxP*	#
M. tuberculosis ML859	$mc^2$6230 derivative; Δ*mmpS4*::*loxP*, Δ*mmpS5*::*loxP*	[5]
M. tuberculosis ML2277	$mc^2$6206 derivative; Δ*smtB-zur*::hyg^R (*rv2358-rv2359* deletion)	This study
M. tuberculosis ML2278	$mc^2$6206 derivative; Δ*smtB-zur*::hyg^R, L5 attB::pML4218 (P_{smtB}::*smtB*), kan^R	This study
M. tuberculosis ML2279	$mc^2$6206 derivative; Δ*smtB-zur*::hyg^R, L5 attB::pML4219 (P_{imyc}::*zur*), kan^R	This study
M. tuberculosis ML2280	$mc^2$6206 derivative; Δ*smtB-zur*::hyg^R, L5 attB::pML4220 (P_{smtB}::*smtB-zur*), kan^R	This study
M. tuberculosis ML1600	$mc^2$6230 derivative; Δ*mbtD*::hyg^R (*rv2381c* deletion)	[6]

S1 Table. Bacterial strains used in this work.

The annotations hyg^R and kan^R indicate that the strain is resistant to the antibiotics hygromycin and kanamycin, respectively. Mutant strains were constructed using either Mtb $mc^2$6230 strain or $mc^2$6206 strain as parent strains as indicated.

#: The construction of these strains will be published elsewhere.
