# Supplementary material for: Comprehensive analysis of iron utilization by Mycobacterium tuberculosis
Source: PLoS Pathog. 2020 Feb 18;16(2):e1008337. doi: 10.1371/journal.ppat.1008337 (PMC7058343; doi:10.1371/journal.ppat.1008337)
Supplement: S2 Table — (PDF) [file ppat.1008337.s008.pdf]

Oligo-nucleotide	Sequence (5' to 3')	Use
Adapter1	CG**ACCACGACCAAACCAAG** (3' C6-TFA-amino modification)	Adapter ligation
Adapter2.x	**3' end of Adaptor-primer** **Index** **Reverse complement of Adapter1**	Adapter ligation
Adapter2.1	**AGTCTCGCAGATGATAAGG****AGACAAGAG****CTTGGTTTGGTCGTGGT**	Adapter ligation
Adapter2.2	**AGTCTCGCAGATGATAAGG****TCACAACTC****CTTGGTTTGGTCGTGGT**	Adapter ligation
Adapter2.3	**AGTCTCGCAGATGATAAGG****ACAGTACCT****CTTGGTTTGGTCGTGGT**	Adapter ligation
Adapter-primer	GTCC**AGTCTCGCAGATGATAAGG**	Junctions amplification
Trans1-r	CCCGAAAAGTGCCACCTAAATTGTAAGCG	Junctions amplification
Trans2-f	CGCCTTCTATCGCCTTCTTGACGAG	Junctions amplification
Nest-1-f	AATGATACGGCGACCACCGAGATCTACACTCTTTCCCTACACGACGCTCTTCCGATCTCGGGGACTTATCAGCCAACC	Hemi-nested PCR
Nest-2.x-r	**Illumina PCR Primer 2.0** **Sample Index** **Illumina Read 2 Sequencing Primer** **Nest-Adaptor primer**	Hemi-nested PCR
Nest-2.1-r	CAAGCAGAAGACGGCATACGAGAT**AAGTAGAG**GTGACTGGAGTTCAGACGTGTGCTCTTCCGATCTGTCA**AGTCTCGCAGATGATAAGG**	Hemi-nested PCR
Nest-2.2-r	CAAGCAGAAGACGGCATACGAGAT**ACACGATC**GTGACTGGAGTTCAGACGTGTGCTCTTCCGATCTGTCA**AGTCTCGCAGATGATAAGG**	Hemi-nested PCR
Nest-2.3-r	CAAGCAGAAGACGGCATACGAGAT**CATGATCG**GTGACTGGAGTTCAGACGTGTGCTCTTCCGATCTGTCA**AGTCTCGCAGATGATAAGG**	Hemi-nested PCR
Nest-2.4-r	CAAGCAGAAGACGGCATACGAGAT**ATCACGAC**GTGACTGGAGTTCAGACGTGTGCTCTTCCGATCTGTCA**AGTCTCGCAGATGATAAGG**	Hemi-nested PCR
Nest-2.5-r	CAAGCAGAAGACGGCATACGAGAT**ACAGTGGT**GTGACTGGAGTTCAGACGTGTGCTCTTCCGATCTGTCA**AGTCTCGCAGATGATAAGG**	Hemi-nested PCR
Nest-2.6-r	CAAGCAGAAGACGGCATACGAGAT**GAGACACA**GTGACTGGAGTTCAGACGTGTGCTCTTCCGATCTGTCA**AGTCTCGCAGATGATAAGG**	Hemi-nested PCR
Nest-2.7-r	CAAGCAGAAGACGGCATACGAGAT**TGTGACCA**GTGACTGGAGTTCAGACGTGTGCTCTTCCGATCTGTCA**AGTCTCGCAGATGATAAGG**	Hemi-nested PCR
Nest-2.8-r	CAAGCAGAAGACGGCATACGAGAT**TGTTCTAG**GTGACTGGAGTTCAGACGTGTGCTCTTCCGATCTGTCA**AGTCTCGCAGATGATAAGG**	Hemi-nested PCR

S2 Table. Oligonucleotides used in this work for DNA sequencing
Indices are underlined.
