# Supplementary material for: Comprehensive analysis of iron utilization by Mycobacterium tuberculosis
Source: PLoS Pathog. 2020 Feb 18;16(2):e1008337. doi: 10.1371/journal.ppat.1008337 (PMC7058343; doi:10.1371/journal.ppat.1008337)
Supplement: S3 Table — (PDF) [file ppat.1008337.s009.pdf]

Oligonucleotide	Sequence (5’ to 3’)	Use
2047-UP-f	AGAGATACTAGTCTGCTGCAACGCCTGGAAG	Gene deletion, PCR validation
2047-UP-r	CTCTCAATTTAAATACCACATCCGCACCTGTCAT	Gene deletion
2047-DN-f	CACACTTAATTAAGAAGCGATCTCAGTCGCTGA	Gene deletion
2047-DN-r	ATATATATGCATGGTGCGCTCGTGCATAGAC	Gene deletion, PCR validation
smtB-UP-f	AGAGATACTAGTGTTCACGAAGATGCCCTGC	Gene deletion, PCR validation
smtB-UP-r	CTCTCAATTTAAATGTTGAGGGGGACGTCACCAT	Gene deletion
zur-DN-f	CACACTTAATTAACTCAGACTGCCGGAGCTAG	Gene deletion
zur-DN-r	ATATATATGCATGACCTCTTCCTGCGGACCTC	Gene deletion, PCR validation
2047-inner-f	GAGACCATCGACGATTTGCA	PCR validation
zur-inner-f	AACATCGGTCTGACCACCGT	PCR validation
gfp-r	TCACCGTCCAGCTCGACCAGGAT	PCR validation
hyg-f	CCTTCACCTTCCTGCACGACTTC	PCR validation
rv2047-EcoR-f	AGAGGAATTCTTAATTAACAGAAAGGAGGTTAATAGTGAGAATCGCCGTCACC	Expression
rv2047-Kpn-r	ATATGGTACCAAGCTTTCAGCGACTGAGATCGCTTC	Expression
smtBup-Eco-f	ATAGGAATTCCTAGCGGACCATAGTCCCAC	Expression
smtB-Kpn-r	AGATGGTACCCACTCATATTGCGTCCTCAC	Expression
zur-Eco-f	AGAGGAATTCCAGAAAGGAGGTTAATAATGAGTGCAGCCGGTGTC	Expression
zur-Kpn-r	ATATAGGTACCCTAGCTCCGGCAGTCTGAG	Expression

S3 Table. Oligonucleotides used in this work for construction of *M. tuberculosis* deletion mutants and for cloning of expression vectors.

Restriction sites are underlined.
