# Supplementary material for: Comprehensive analysis of iron utilization by Mycobacterium tuberculosis
Source: PLoS Pathog. 2020 Feb 18;16(2):e1008337. doi: 10.1371/journal.ppat.1008337 (PMC7058343; doi:10.1371/journal.ppat.1008337)
Supplement: S4 Table — (PDF) [file ppat.1008337.s010.pdf]

Plasmid	Components and properties	Source or reference
ΦMycoMarT7	*Himar1* transposon, kan^R	[7]
pML2714	pUC origin; pAL5000ts; P_{hsp60}::*cre*; P_{imc}::*pamcherry1m*; *aph*; 7613 bp	[8]
pML4211	ColE1 origin; *int*; L5 *attP*; *aph*; P_{imyc}::*rv2047c*; 7730 bp	This study
pML4218	ColE1 origin; *int*; L5 *attP*; *aph*; P_{smtB}::*smtB*; 5753 bp	This study
pML4219	ColE1 origin; *int*; L5 *attP*; *aph*; P_{imyc}::*zur*; 5550 bp	This study
pML4220	ColE1 origin; *int*; L5 *attP*; *aph*; P_{smtB}::*smtB-zur*; 6142 bp	This study
pML3605	pML2424-derivative; pUC origin; pAL5000ts; *sacR*; *sacB*; *tdTomato*; up_*rv2047c*_hom; *loxP*-P_{smyc}::*gfp-hyg-loxP*; down_*rv2047c*_hom; 10850 bp	This study
pML3606	pUC origin; pAL5000ts; *sacR*; *sacB*; *tdTomato*; up_*smtB*_hom; *loxP*-P_{smyc}::*gfp-hyg-loxP*; down_*zur*_hom; 11127 bp	This study
pML3001	pUC origin; pAL5000ts; sacR; sacB; *tdTomato*; up_*mmpL4*_hom; *loxP*-P_{smyc}::*gfp-hyg-loxP*; down_*mmpL4*_hom; 11579 bp	*
pML3003	pUC origin; pAL5000ts; sacR; sacB; *tdTomato*; up_*mmpL5*_hom; *loxP*-P_{smyc}::*gfp-hyg-loxP*; down_*mmpL5*_hom; 11472 bp	*

S4 Table. Plasmids used in this work.

Up- and downstream homologous sequences of genes are subscripted as up and down. "Origin" denotes origin of replication. The genes *hyg* and *aph* confer resistance to hygromycin and kanamycin, respectively.

* The construction of these plasmids will be published elsewhere.
