# Supplementary material for: Comprehensive analysis of iron utilization by Mycobacterium tuberculosis
Source: PLoS Pathog. 2020 Feb 18;16(2):e1008337. doi: 10.1371/journal.ppat.1008337 (PMC7058343; doi:10.1371/journal.ppat.1008337)
Supplement: S5 Table — (PDF) [file ppat.1008337.s011.pdf]

| Library | Medium (Iron-free 7H9 agar, Tyloxapol, Kan) | Mapped reads | Template counts | NZmean | #TA sites hit | Density (%) | NCBI BioSample accession no. |
|---|---|---|---|---|---|---|---|
| Fe-cMBT_rep1* | 500 ng/ml Fe-cMBT | 9,652,517 | 697,522 | 12.3 | 56,724 | 76.2 | SAMN12913093 |
| Fe-cMBT_rep2 | 500 ng/ml Fe-cMBT | 2,305,805 | 283,558 | 5.7 | 49,620 | 66.7 | SAMN12913094 |
| High Fe-MBT_rep1* | 250 ng/ml Fe-MBT | 8,993,212 | 764,055 | 13.8 | 55,351 | 74.4 | SAMN12913095 |
| High Fe-MBT_rep2 | 250 ng/ml Fe-MBT | 2,231,988 | 393,946 | 7.9 | 50,169 | 67.4 | SAMN12913096 |
| High Fe-MBT_rep3 | 250 ng/ml Fe-MBT | 4,173,269 | 538,618 | 10.0 | 53,894 | 72.4 | SAMN12913097 |
| Low Fe-MBT_rep1* | 50 ng/ml Fe-MBT | 13,288,416 | 873,980 | 15.8 | 55,381 | 74.4 | SAMN12913098 |
| Low Fe-MBT_rep2 | 50 ng/ml Fe-MBT | 9,345,606 | 839,566 | 15.0 | 56,018 | 75.3 | SAMN12913099 |
| Hemin_rep1* | 20 µM hemin | 11,218,510 | 587,959 | 10.8 | 54,654 | 73.4 | SAMN12913100 |
| Hemin_rep2 | 20 µM hemin | 7,952,349 | 676,152 | 12.3 | 55,001 | 73.9 | SAMN12913101 |
| Hemin_rep3 | 20 µM hemin | 6,281,407 | 608,542 | 11.1 | 55,065 | 74.0 | SAMN12913102 |
| Hemoglobin_rep1* | 5 µM hemoglobin | 10,724,437 | 597,622 | 10.6 | 56,181 | 75.5 | SAMN12913103 |
| Hemoglobin_rep2 | 5 µM hemoglobin | 8,626,564 | 963,195 | 17.0 | 56,640 | 76.1 | SAMN12913104 |
| Hemin-MBT_rep1* | 20 µM hemin, 250 ng/ml Fe-MBT | 13,721,967 | 788,621 | 14.0 | 56,230 | 75.6 | SAMN12913105 |
| Hemin-MBT_rep2 | 20 µM hemin, 250 ng/ml Fe-MBT | 10,408,244 | 921,888 | 16.2 | 56,995 | 76.6 | SAMN12913106 |
| **Average** | | **8,494,592** | **681,087** | **12.3** | **54,851** | **73.7** | |

**S5 Table. Statistical analysis of the *M. tuberculosis* transposon libraries used in this work.**
The Mtb Δ*mbtD::hyg*$^r$ (ML1600) genome contains 74,410 TA sites. NZmean denotes the average template count at non-zero TA sites. Density denotes the fraction of TA sites with at least one insertion.
* The data sets were used for HMM analysis.
