# Supplementary material for: Comprehensive analysis of iron utilization by Mycobacterium tuberculosis
Source: PLoS Pathog. 2020 Feb 18;16(2):e1008337. doi: 10.1371/journal.ppat.1008337 (PMC7058343; doi:10.1371/journal.ppat.1008337)
Supplement: Attachment — Submitted filename: MtbFe_Rebuttal_v09.pdf [file ppat.1008337.s016.pdf]

Rebuttal – Manuscript PPATHOGENS-D-19-02065

Manuscript title: Comprehensive analysis of iron utilization by *Mycobacterium tuberculosis*
Authors: Zhang et al.

Part I - Summary

We thank all three reviewers for their positive comments and the constructive criticism of our manuscript. We have addressed their critiques as described below.

Part II – Major Issues: Key Experiments Required for Acceptance

None requested.

Part III – Minor Issues: Editorial and Data Presentation Modifications

Reviewer #1:

1) P.6, line 104 - presumably this should read "...were generated IN media containing...
2) P.7, line 129 - should read "...which exhibited A statistically significant....
3) P.8, line 157 - should read "...the electron transfer flavoprotein FixAB... (i.e. "flavoprotein" rather than "flavoproteins")
4) P.9, line 177 - should read "...previous experiments WHICH SHOW THAT neither the...."
5) P.9, line 198 - change "effect" to "affect"
6) P.15, line 306 - should read "...significant or complete growth defect of Mtb only.... (i.e. "defect" rather than "defects")
7) P.15, line 321 - should read "One example IS the hem genes..."
8) P.15, line 324 - should read "...standard Mtb growth MEDIUM containing iron salts..."
9) P.17, line 368 - delete the first "the" to read "...was confirmed by the absence of any growth defect..."
10) P.19, line 401 - should read "...experiment is in conflict WITH the observation that Mtb..."
11) P.19, line 413 - should read "...to converge on THE cell surface of Mtb [23]."
12). P.20, line 428 - correct the spelling of "tetrapyrrolE" (i.e. add an e on the end of the word).
13) P.21, line 451 - should read "...compared with THE wt strain, indicating that Zur..." (i.e. insert "THE" and insert a comma after "strain")
14) P.21, line 454 - should read "...repressor which IS not known to activate genes."

We thank the reviewer for carefully reading our manuscript and pointing out the mistakes. We made all edits as suggested.

Reviewer #2:

1) Line 120-122, the authors imply that hemin rescues irtAB Tn mutants. However, the TN insertion profile of the library grown in hemin + MBT looks very similar to the profile of the TN library grown in MBT. Thus, it would seem that the irtAB could contribute to the MBT intoxification phenotype. How is the rescue of a phenotype quantified based on Tn insertion count?

We noticed the siderophore-poisoning pattern for *irtA/irtB*, but it is not as pronounced as for *mmpS5/mmpL5* or for *rv2047c and rv0455c.* Hence, we initially did not describe this observation. Now, we rephrased this paragraph and state that: "Although *irtA* and *irtB* were found to be not essential in the presence of hemin and hemoglobin, adding high concentrations of MBT in the presence of hemin significantly reduced the fitness of Mtb mutants with insertions in *irtA* and *irtB* indicating that reduced uptake of cMBT and/or MBT across the inner membrane is also toxic for Mtb (Figs 3B, 5B, S6 Table)."

2) Several genes are identified as being siderophore toxic secretion mutants, a concept that has been widely promoted in the Mtb research community. The identification of several genes that are previously reported to participate in efflux of MBT in these studies support this hypothesis. Were any genes identified that had a similar phenotype with hemin (i.e. low Tn insertion in hemin and hemin + MBT)

Indeed, we identified 7 genes that showed a heme poisoning pattern: not essential with cMBT/high MBT, essential with hemin and with hemin+MBT. However, 6 of those genes were also classified as essential or "growth defect" with low MBT. In addition, none of these genes encoded transport proteins. Thus, it is questionable whether a heme poisoning effect exists in Mtb. This is now described in an additional paragraph on p. 15.

3) The authors identified a number of genes that lacked a defined function or a function not specifically associated with iron uptake or siderophore export. The authors do not consider that a proteins function may be directly tied to the source of iron. It would be interesting to know if gene products that require iron for function were identified in the differential iron source screen.

Rv0338 is predicted to bind an iron-sulfur-cluster and is the only such gene with differing requirements by Mtb under different iron conditions. *Rv0338* is essential for growth of Mtb in medium with low MBT, hemin and hemoglobin and *Rv0338* is not essential in medium with cMBT or high MBT.

4) The results presented in Figure 7 and Page 12 did not include evaluation in the presence of hemin + MBT. This is a primary experiment to define a siderophore secretion phenotype mutant.

We agree with the reviewer that examining the growth of the Δ*mmpS4/mmpS4*, Δ*mmpL4/L5* and Δ*rv2047c* mutants in medium with heme+MBT would reproduce the conditions which we used in the TnSeq experiment to identify siderophore poisoning. Comparison of a Tn mutant at different growth conditions (media with hemin and media with hemin +MBT) is necessary in the TnSeq experiment since no parent strain is available. However, comparison with the parent strain in media with hemin or MBT is sufficient to demonstrate siderophore toxicity for isogenic mutants.

5) Line 104. I think the authors meant to say "generated on media" not "generated media.

This statement was rephrased.

6) Line 310 carboxymycobactin and mycobactin should be abbreviated

Done as suggested.

Reviewer #3:

1. Intro – A brief description of MBT vs cMBT might be helpful to non experts

We added a description of MBT vs cMBT as requested (ll. 43-46). " These siderophores share a common core structure determining their iron (III) chelating properties, but the secreted cMBTs have significantly shorter fatty acid chains and are, hence, more hydrophilic than the MBTs which are completely insoluble in water and are, therefore, membrane associated [6,8]."

2. Line 47-49 is this speculation or known?

References in support of the statement that siderophores could be shared among Mtb cells were added: "These siderophores could be shared among Mtb cells in areas of high bacterial loads such as in macrophages or in the lungs of patients with active TB via intracellular [14] or extracellular vesicles [15], respectively, enabling efficient iron uptake."

3. Line 89 vs line 102 – be consistent with units even though plates and broth are different

The molar concentrations of the different iron sources were added.

4. Line 146-152 awkward wording

These sentences were rephrased.

5. Line 201-239 – How is this section different from the next section? These should be merged or differentiated

These sections are now differentiated. The first chapter in this section is now entitled "Siderophore poisoning of Mtb" and now also contains a description of the observation that the *irtA/irtB* genes show a siderophore poisoning pattern as requested by reviewer 2. It is obvious that siderophore poisoning is not only caused by blocking siderophore secretion but also by other mechanisms. The second paragraph is entitled "Roles of the MmpL4/MmpS4 and MmpL5/MmpS5 efflux systems in siderophore-mediated iron utilization by *M. tuberculosis*".

6. Line 241 title is misleading in describing what is actually written in the section.

The title of this paragraph was slightly changed to account for the fact that we did not measure siderophore secretion directly. The title is now: "Roles of the MmpL4/MmpS4 and MmpL5/MmpS5 efflux systems in siderophore-mediated iron utilization by *M. tuberculosis*".

7. Line 299-301 – This text appears to associate smtB to hemoglobin and zur to heme but that is not supported by the data

The paragraph describing the results obtained with the Δ*smtB-zur* double mutant was edited to enhance clarity. Our data clearly show that Zur is required for heme utilization by Mtb and that <u>both regulators</u>, Zur and SmtB are required for growth of Mtb on hemoglobin as an iron source.

8. Line 420. Have the authors determined how log heme is stable in agar? Perhaps heme degradation accounts for the discrepancies across different bodies of work?

We did not determine the stability of heme in agar. Heme might be degraded to some extent during the extended growth of Mtb on agar plates. However, there is sufficient heme available to support the

growth of Mtb for several weeks. In our previous study, 20 µM hemin was sufficient to support the growth of Mtb on the agar plates for at least four weeks at 37°C (*PLoS Pathog* **9**: e1003120; 2013). Experiments are currently underway to examine the reasons for the discrepancy of the roles of PPE36 and PPE37 in different studies. This is a considerable effort beyond the scope of this study.

9. Line 449-451. Under what condition was this done and was this relevant/comparable to iron-deficient conditions that this work examines?

The experimental details of these studies are published. In the paper by Maciag et al. Mtb was cultured in standard Middlebrook 7H9 (Difco) medium (i.e. Iron-rich, Zinc-rich) supplemented with ADN (2% glucose, 5% bovine serum albumin, 0.85% NaCl) and 0.05% Tween 80 for the DNA microarray experiments (*J Bacteriol* **189**: 730-; 2007). The authors of this study concluded that Zur is a Zinc-responsive repressor since 32 genes were up-regulated and no genes were down-regulated in the *zur* mutant. In our growth assays, the growth of the *zur* mutant was unaffected by the iron concentration (S6 Fig). This result is consistent with the previous observation that Zur is specifically responsive to Zinc ions but not to other metals ions (such as iron) (*J Bacteriol* **189**: 730-; 2007). Taken together, we assume that the iron concentration does not significantly change the Zur-controlled transcriptional profile in Mtb.

10. The discussion could be improved by some attention to big biological questions on iron acquisition and how it impacts mtb pathogenesis. Like where would mtb preferentially use heme vs using siderophores?

A short paragraph was added to the end of the discussion to describe which iron sources are available for Mtb during different stages of the infection.

11. Line 479 – how were these obtained?

We thank the reviewer for pointing out this oversight. The siderophores were purified from *Mycobacterium bovis* BCG and *Mycobacterium smegmatis* and were provided by Dr. Colin Ratledge. This is now mentioned in the Materials and methods section and in the Acknowledgements.

12. Fig 6 and 7 – legend and figure are switched

Thank you for pointing this out. This error was corrected.

13. Fig 7 –7D the mmpL5 growth lag is confusing. How this relates to the description that this gene is "required" should be discussed in more detail

The importance of MmpL5 is obvious in the significant growth delay of the single *mmpL5* deletion mutant which is absent in the single *mmpL4* deletion mutant. This is easily explained by the much higher expression levels of MmpL5 compared to MmpL4 and the resulting increased susceptibility of the Mtb mmpL5 mutant to the toxicity of MBT through impaired siderophore secretion.

The confusion may arise from the fact that the *mmpL5* gene was classified as "essential" in the HMM analysis of the TnSeq data, but the mmpL5 deletion mutant still has residual growth in liquid medium. It is possible that this difference is caused by different growth requirements on solid medium compared to liquid medium. The more limited nutrient availability on agar plates leads to more stringent growth conditions and may enhance growth defects of mutants. Considering the growth competition in the pooled library on plates, the *mmpL5* transposon mutants likely fail to compete with the many other

viable Tn mutants. Thus, the *mmpL5* gene was assessed as "essential" for Mtb grown with high MBT on solid medium in the TnSeq experiments.

Other changes:

We carefully revised the entire manuscript in addition to the changes in response to the reviewer's comments to improve readability. All changes are marked in a Word file named "_MarkedRevisions.docx".
